# Novel Microwave Torso Scanner for Thoracic Fluid Accumulation Diagnosis and Monitoring

**DOI:** 10.1038/s41598-017-00436-w

**Published:** 2017-03-22

**Authors:** S. Ahdi Rezaeieh, A. Zamani, K. S. Bialkowski, A. M. Abbosh

**Affiliations:** School of ITEE, The University of Queensland, St Lucia, 4072 Brisbane Australia

## Abstract

Thoracic fluid accumulation is one of the significant and early-stage manifestations of fatal diseases, such as lung-cancer, liver-failure and congestive heart-failure. Currently, computational-tomography (CT)-Scan is the most widely used tool for the detection of thoracic fluid. Yet, it is unable to detect small amounts of fluid, has ionizing radiation and lacks mobility. On the other hand, microwave imaging systems have emerged as an accurate and portable complementary diagnostic tool. However, there is a lack of a complete clinical platform that can fulfill the requirements of accurate and reliable imaging. Therefore, a microwave torso scanner that is designed to meet those requirements is presented. In this system, two elliptical-arrays of microwave antennas (sensors) transmit signals towards the torso and collect the back-scattered signals. The captured signals are then processed by a frequency-based imaging algorithm to form microwave images that display a possible accumulated fluid. The system successfully detects and localized small volumes (3 mL) of fluid injected at different places inside a torso-phantom. As preparations for future clinical trials, the system is tested on healthy subjects to define the threshold range of healthy scenario images.

## Introduction

Lung cancer, liver failure and cardiovascular diseases (CVDs) are among the illnesses that threaten the wellbeing of modern societies due to the increase in the consumption of tobacco products, alcohol, and lack of exercise besides genetic inheritances. According to the fact sheet that has been released by World Health Organization (WHO) in January 2015^1^, around 17.5 million people have lost their lives due to CVDs. This number represents about 31% of the total deaths in the world. Based on these data, more than 75% of these mortalities take place in middle or low income countries, where there is limited access to medical diagnostic tools, such as magnetic resonance imaging (MRI) or CT-scan. One of the common signs of the aforementioned diseases is the accumulation of fluid inside the torso area (inside or around the lungs). As a result, the lungs are prevented from exchanging the CO_2_ directed from the heart with oxygen inhaled by the lungs causing fatal consequences if not treated properly and timely^2, 3^. Therefore, the early stage detection and monitoring of the thorax area is the key to early medical intervention and thus prevention of the health decline or even death. This aim is of great importance for the welfare of the society, and is essential to relieve the associated burden from the healthcare system.

Great efforts have been invested in finding new diagnostic tools for the early detection of those diseases in recent decades. Currently, CT-scan are the most widely used devices for the detection of the fluid, which has a high water content, inside the thorax area. This technology, which was proposed in 1967, utilizes computational techniques to analyze several X-ray images and produce a more accurate image of the investigated area of the body. Despite its impressive performance in terms of providing clear images, there are serious concerns regarding its safety as it imposes rays that are extremely higher than the conventional X-rays, and therefore, cannot be used by certain categories of patients, e.g. pregnant women, and more importantly, cannot be used frequently. This latter shortfall is a serious drawback considering the fact that a monitoring tool is needed in the rehabilitation process to monitor the fluid level with medication.

To address the requirement for a safe and low-cost device that can be used both for the diagnosis and monitoring purposes, the prospect of utilizing microwaves for the detection of thoracic fluid accumulation inside was proposed in 1973^4^. It is based on the fact that reflected microwave signals experience amplitude and phase variations depending on the dielectric properties of the imaged medium. Compared to the dielectric constants (*ε*_*r*_) of the tissues existing at torso area, such as the skin (*ε*_*r*_ = 41.4), muscle (*ε*_*r*_ = 55), fat (*ε*_*r*_ = 5.4), lungs (*ε*_*r*_ = $$22)$$, liver (*ε*_*r*_ = 46.8) and heart (*ε*_*r*_ = 59.8), water has considerably higher dielectric constant (80.4), which consequently creates a higher reflection that is distinct from others, and hence can be used as a sign of the presence of fluid (*ε*_*r*_ values are calculated at 900 MHz). Based on this analysis, different methods that uses the amplitude or phase of the reflected or transmitted signals as a source for water detection have been proposed in recent years^5–9^. To determine the practicality of the proposed ideas, several experiments were conducted^5, 6^. In these experiments, single antenna (microwave sensor) or array of antennas are connected to a vector network analyzer (VNA) and used to transmit microwave signals towards the torso and receive the reflected signals. Early studies conducted on phantoms and dogs^10^ revealed that while the changes in amplitudes of the reflected signals are consistent with the changes in the level of the accumulated fluid, phase variations provide a bigger difference with respect to the changes in the fluid volume. By utilizing this technique, an applicator was designed and attached directly to the lungs of a human mannequin^7^. It was shown that by injecting water inside the lungs (in the form of sponges), the phase changes by up-to 2.5 degrees. In another approach, the properties of the transmitted signal was used to estimate the average dielectric constant of the medium^8^. In that system, one sensor was used to transmit the signal and several sensors were used to collect the transmitted signals. The obtained dielectric constant number is then compared with the databased of a healthy lung to determine the presence of fluid. Besides microwave techniques, there have been several other methods investigated for the possible detection of fluid accumulation inside lungs, such as acoustic techniques^11^, bioimpedance spectroscopy^12^, radiometry^13^ and electrical impedance tomography^14^. Nevertheless, studying the abovementioned literature reveals that while there has been considerable progress in signal processing, imaging and detection methods, there is a lack of comprehensive study on the system’s hardware design aspects which are the main element in obtaining reliable raw data for accurate imaging in a clinical environment. Consequently, there have been limited success in building a clinical platform that can be tested in realistic and clinical scenarios except for the recent study^15^. However, while that system has proven to be successful in detecting small volumes of fluid, it has limitations in determining the exact fluid position, which is vital in cases where a minimal invasive biopsy is needed to determine the type of oedema causing the fluid accumulation. The limitation of the fore stated system^15^ comes from the fact that it cannot comply with the requirements of microwave imaging techniques that dictate fixed and known distances to the imaging body besides using a linear array configuration that can only scan the rear side of the torso. Consequently, it cannot detect fluid accumulated in deep positions.

To address the abovementioned limitations and enhance the quality of diagnosis in terms of detecting and localizing fluid at any depth, a microwave torso scanner with emphasis on the whole system design is proposed in this paper. This system is designed in a shape of a doughnut chamber. It includes a base section that is installed to a clinic wall or ambulance and an opening fragment that operates as a door that is closed when the patient is positioned inside the scanner. Two arrays of twelve antennas are accommodated inside the chamber in the vicinity of the surface of the scanner to transmit and receive microwave signals towards and from the torso, respectively. The system is designed in a shape that the antennas are all positioned in a fixed and known distance as needed for accurate imaging. To accommodate those two antenna arrays in close vicinity of each other and within the limited dimensions around the human torso, the theory of metamaterial structures is utilized and an epsilon-negative (ENG) metamaterial unit-cell loaded Yagi-antenna is designed. The antenna elements of the two arrays are connected to a switching system, which facilitates the electronic scanning of the torso by transceiving signals via a portable vector network analyzer. The captured signals are stored and then processed using a suitable laptop which includes the processing and image creation algorithms. The system was successfully tested on a realistic size artificial phantom to detect small amounts of fluid both in shallow and deep positions. To verify the capability of the system in tracking the exact position of the fluid, it was displaced in different positions and the obtained results are presented. Several scenarios using different system configurations were tested to analyze the success rate of the system in handling various cases. As a first step in defining a global database that can be used as a reference baseline for the detection purpose, six healthy male and female subjects with different body sizes were tested. It is found that there is a considerably meaningful similarity in the intensity of the scattered signals from different healthy cases indicating a feasibility of this system for future clinical trials.

### Torso Scanner System

One of the main limitations associated with the detection methods that utilize variations in the amplitude or phase of reflected or scattered signals as the only detection measure is the lack of defining a real-time reference for the data, i.e. missing a threshold for the correct decision, healthy or otherwise, to be made. While one might argue that a reference database can be built for any individual that reference changes dramatically with time because it is very sensitive to any variation in the human body or the environment of measurements. The only credible reference is the measurements at the moment of scanning but that is obviously not possible as data needs to be compared for the same subject at a healthy and then suspected status. This limitation was partially addressed by utilizing the symmetry between the shapes of the left and right side lungs^15^. In that system, a threshold is defined by subtracting the scattering profiles of the two lungs from each other in the healthy case. In the detection process, the presence of fluid is detected if the scattering profile is stronger than the defined value in the differential image. However, the accuracy of detection is restricted by the imperfect symmetry between the left and right lungs due to the presence of the heart in the left side. Studying the structure of the torso reveals that the heart is not present in the highest and lowest sections. These regions host very similar tissue types with of course different masses; thus, they are expected to possess similar, yet, not identical scattering profiles. Moreover, due to gravity, any accumulated fluid inside the torso tends to gather inside or around the lower sections of the lungs. Therefore, fluid accumulation at the lower region can be identified by comparing the scattering profiles of the upper and lower regions to that of a healthy case.

**Figure1:**
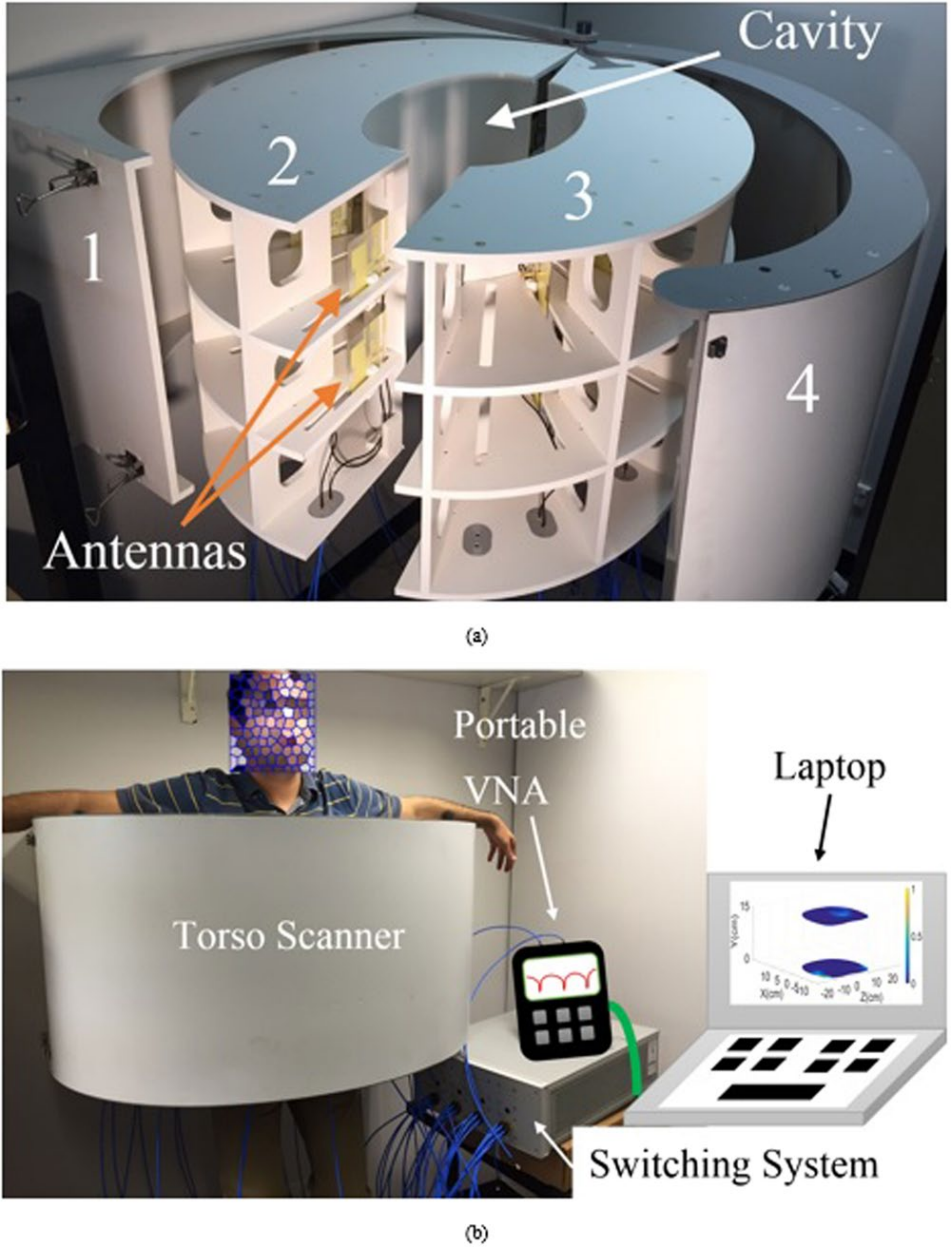
(**a**) Dismantled presentation of the proposed torso scanner installed on a wall. (**b**) Measurement setup with human subject inside the scanner.

A system that can scan the upper and lower regions of the torso at the same time is designed, fabricated and tested. The dismantled structure of the proposed scanner is depicted in Fig. 1(a). The outer layer of the system is designed in a semi-doughnut chamber shape to accommodate the antenna arrays. On the other hand, the inner section of the system, cavity, is designed in an elliptical shape to form similar structure to that of the human torso. It is built using expanded Polyvinyl chloride (PVC) with properties that are radiofrequency (RF) transparent, and therefore, it does not affect the radiation performances of the antennas. The system includes four compartments; the first part is the installation base designed to assist in fixing the system on a clinic wall. The second and third compartments are the upper and lower elliptical flanges forming antenna nests that have several slots with equal angles with respect to the center of the cavity, while the fourth compartment is the cover case of the system. To build the microwave based torso scanner, the system parts are combined together and the antennas are installed inside the designated slots and are fixed using plastic clamps. Therefore, a fixed distance from the scanning face of the scanner (cavity) is achieved. The antennas, which are comprehensively explained in the supplementary file, are then connected to a switching system to electronically scan the circumference of the torso. The switch network is formed using a Keysight L4491A microwave switch platform^16^. The switching platform is connected to the ports of a Keysight N9923A FieldFox vector network analyzer (VNA)^17^. The switching platform is configured with five 87106B-T24 SP6T multiport switches to form a virtual SP24T switch, and thus each of the 24 antennas can be connected to the VNA one at a time. The VNA generates the required microwave signal and measures the frequency response of the connected antenna between 0.5 and 2 GHz. During the experiments, the patient is positioned inside the cavity and the torso is scanned by each of the antennas. Controlling this whole system and saving the data are done by a Python script, which interfaces a laptop to the VNA and the switches via Ethernet and USB connections.

### Safety Considerations

**Figure 2:**
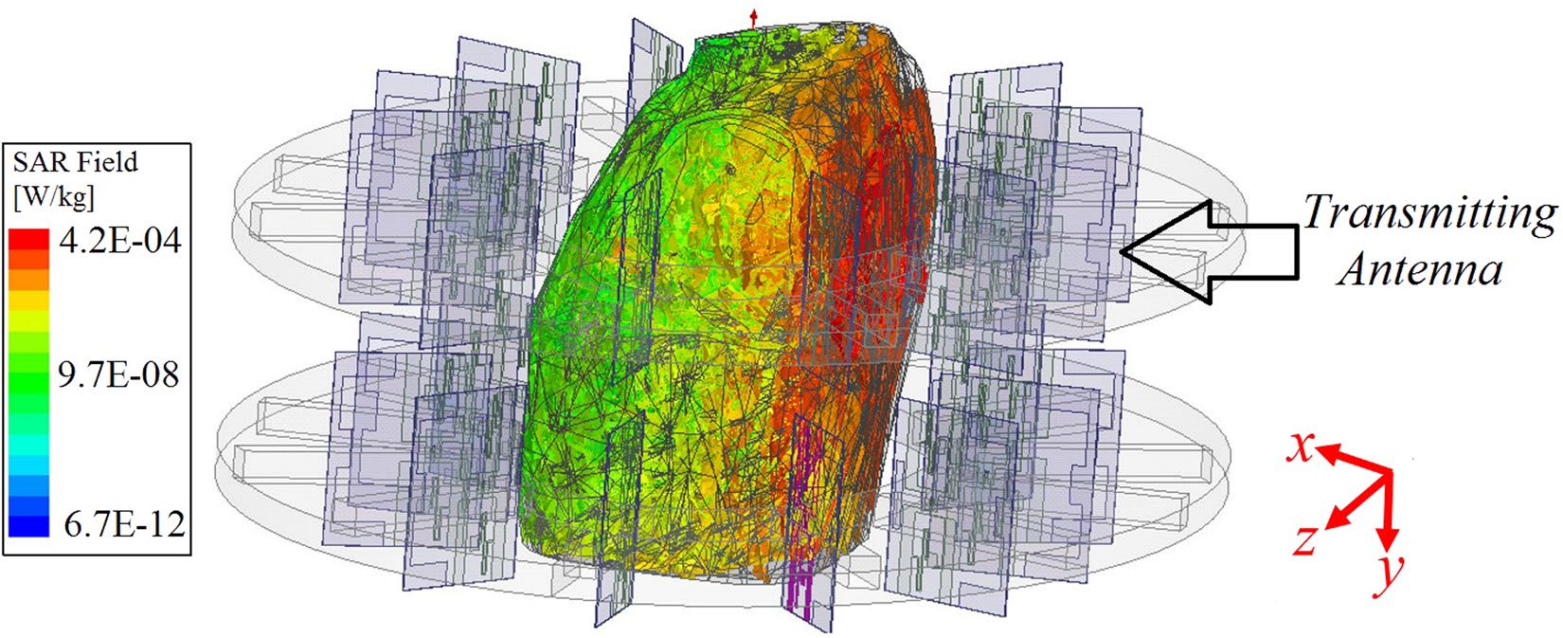
Specific absorption rate distribution in the torso when the antenna at center radiates.

To verify the safety of the system before testing its capabilities, the specific absorption rate (SAR) is calculated in the simulation environment. SAR refers to the energy absorption rate by the human body when exposed to electromagnetic fields. As shown in Fig. 2, the whole system is simulated in Ansys Electronic Desktop (AEDT). To that end, a realistic computational three dimensional (3-D) phantom with more than 300 organs and resolution of 2 mm is placed inside the proposed scanner. The antennas are fixed at a 1 cm distance from the cavity. Considering that SAR value reduces with distance, the closest antenna to the chest wall, which is specified in Fig. 2, is excited. In order to comply with the existing microwave imaging system, the antenna is excited at 0.9 GHz using a power level of 1 mW (0 dBm) on an average tissue mass of 10 grams. It should be noted that the utilized power level is considerably lower than the power used by current mobile phones, which is between 0.6–3 W. As seen from Fig. 2, maximum SAR levels occur at the skin and outer layer of the torso and SAR values decrease with distance and towards the front side of the torso. The calculated SAR value is around 0.004 [W/kg] which is much lower than 0.4 [W/kg] defined by Federal Communications Commission (FCC)^18^ as the safe level for medical diagnostic applications. Hence, the proposed torso scanner can be safely tested on phantoms and human subjects.

### Phantom

**Figure 3:**
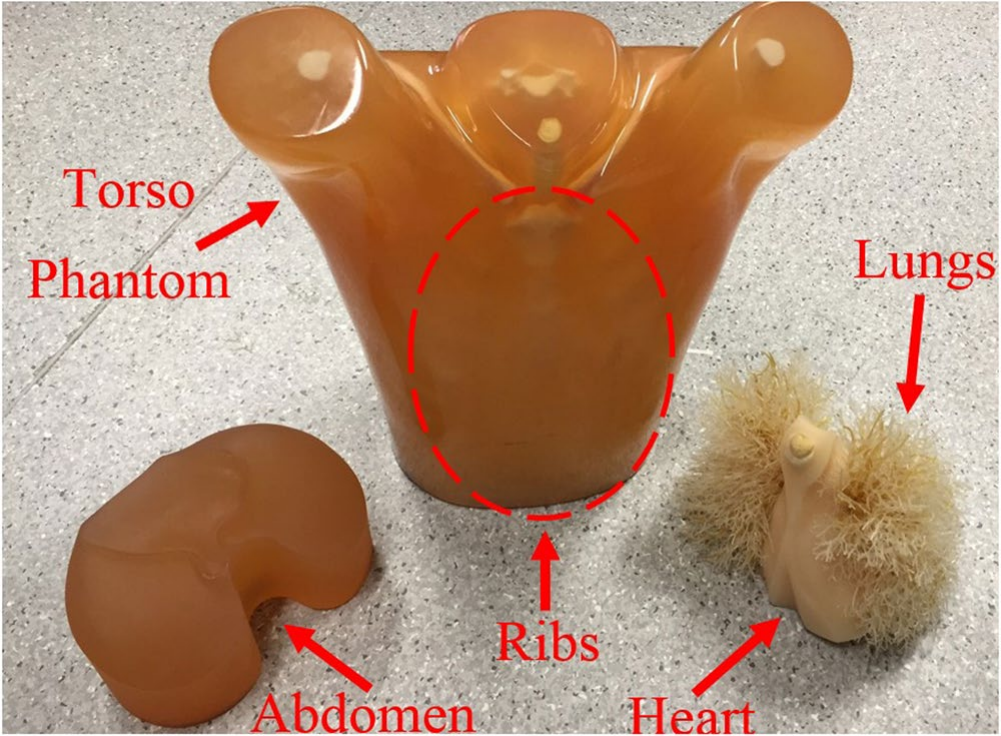
The utilized realistic size torso phantom with disassembled inner organs.

To study different cases of fluid accumulation, the system is tested on a torso phantom that has exact physical measures of an average human torso and is comprised of lungs, heart and abdomen block in addition to complete thorax ribs and muscle tissues (See Fig. 3). The phantom size is 43 × 40 × 48 cm^3^ with chest circumference of 94 cm, and the soft tissues of the phantom are fabricated from polyurethane while the synthetic bones are made using epoxy resin. It should be noted that the utilized phantom does not identically mimic the dielectric properties of the human torso. However, it provides different dielectric constant values for muscles, lung tissues and ribs which provides a heterogeneous medium for microwave imaging. Moreover, compared to existing torso phantoms that are widely used for SAR and torso imaging^7^, which are generally empty shells with homogeneous tissue mimicking liquid, the utilized phantom provides the most complex structural environment and is the closest available to the real scenario.

### Potential Data Acquisition Methods

**Figure 4:**
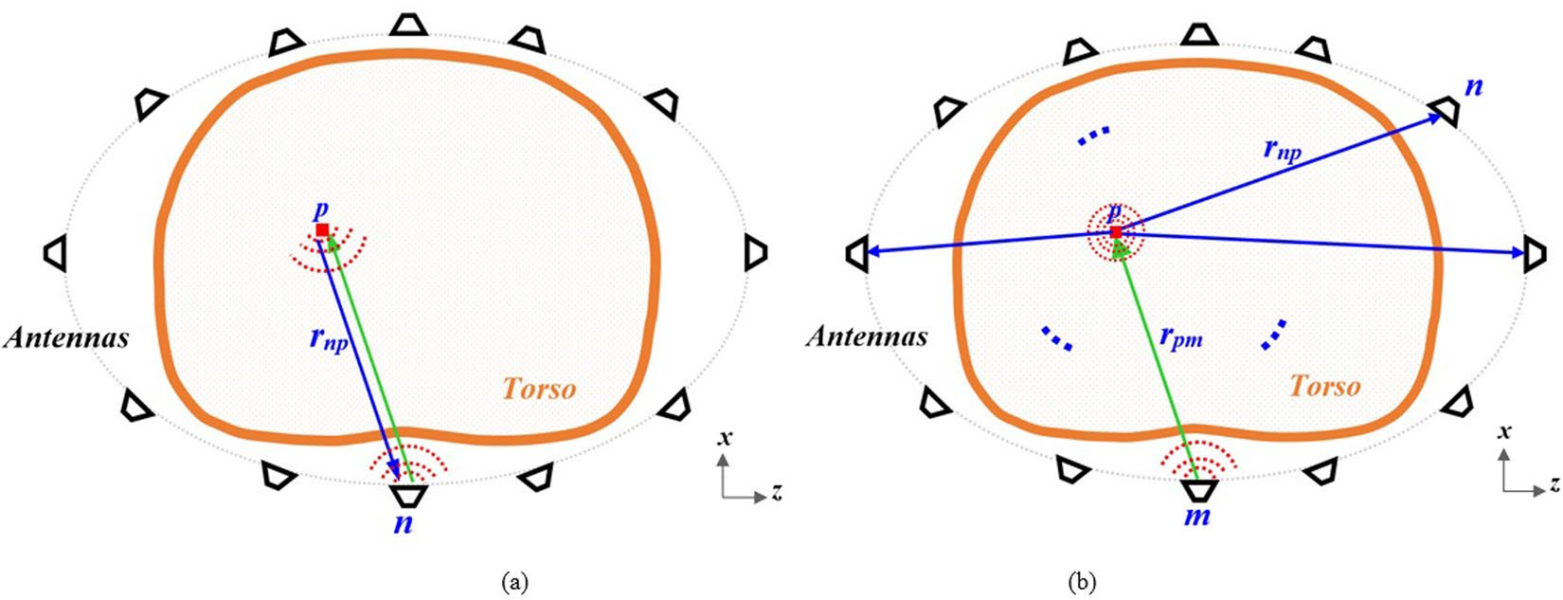
Scattering signal acqusition in (**a**) mono static and (**b**) multistatic configurations.

To verify capability of the system in detecting accumulated thoracic fluids, several test setups are considered. In general, two configurations can be used to acquire back scattered signals from torso; monostatic and multistatic. As shown in Fig. 4(a) and (b), in the monostatic approach, the antennas are individually used for both transmitting and receiving signals, while in the multistatic setup, each of the antennas are used as a transmitter while all the others are used to collect scattered signals. It is noted that *r*_*mp*_ is the distance from the transmitting antenna to the target and the *r*_*np*_ is the distance from the target to the receiving antennas. This procedure continues till a full scan is completed by all the antennas in the array. Multistatic configuration requires larger number of switches compared to the mono-static scenario, however, it provides much more information that increases the accuracy of the detection. To verify the abovementioned notions and study the accuracy of the system and its detection capabilities, both of the configurations are investigated in this paper.

## Results

### Mono-Static Measurements

**Figure 5:**
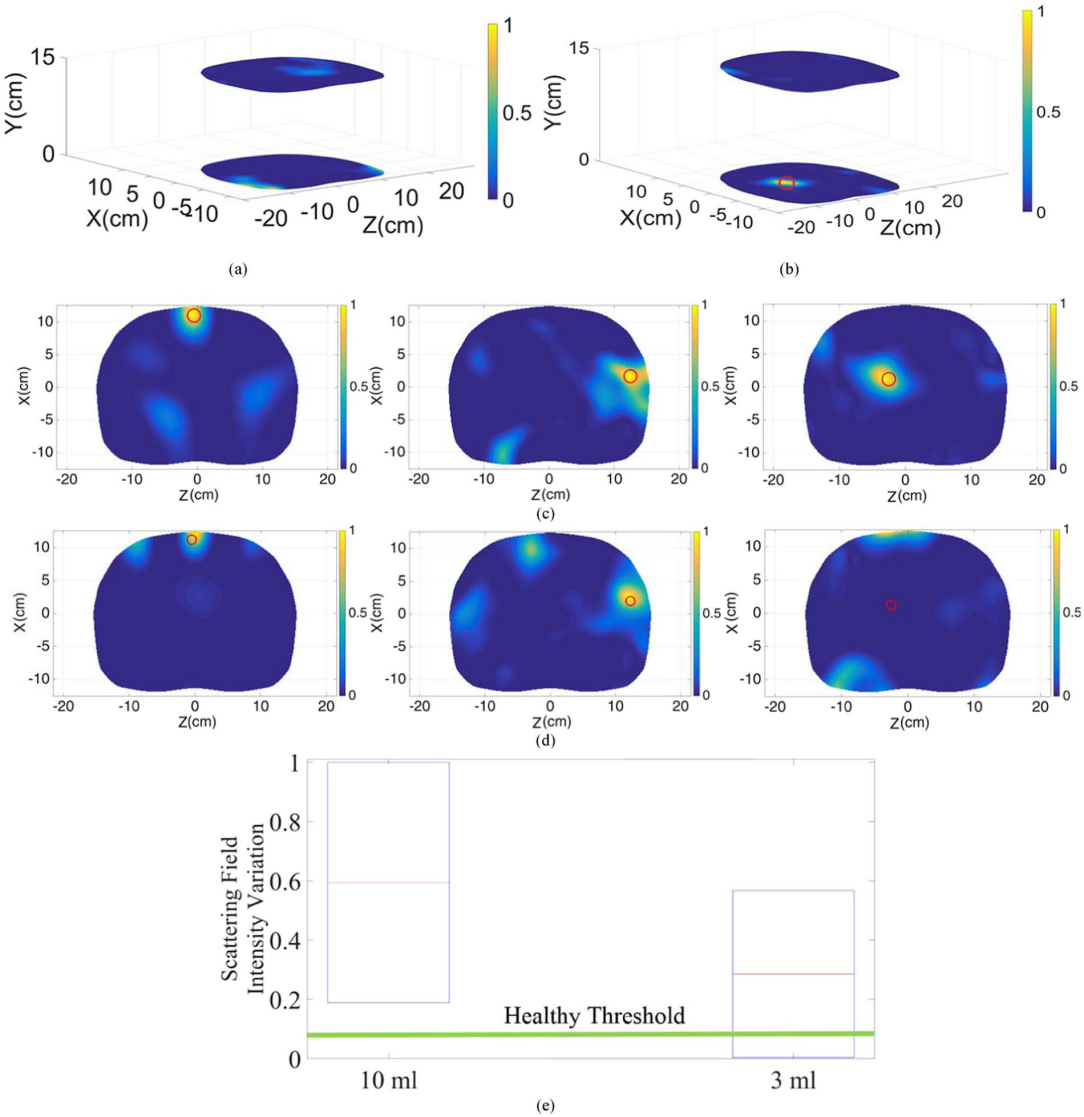
Reconstructed image of the upper and lower regions of the torso in (**a**) healthy case and (**b**) unhealthy case with accumulated fluid inside the thorax area. Subtracted images of the upper and lower regions at three different positions; front side of the torso left), behind ribs middle) and deep inside thorax right) with (**c**) 10 mL and (**d**) 3 mL injected water. (**e**) Scattering field intensity variation diagram for 10 mL and 3 mL inserted water compared to the healthy threshold.

To calibrate the system and remove the amount of inner reflections of the antennas facing each other with the system’s cavity, the system is operated when there is no phantom inside the system and the obtained signals are subtracted from the back scattered signals from torso/human subjects in the subsequent experiments. The experiments were done in uncontrolled environment. This is specifically done to mimic realistic scenarios where temperature of the room or the devices are neither controlled nor monitored. To test the liability of the switching system and the VNA, the experiments were repeated several times to ensure the validity of the obtained data. It was realized that time and temperature has negligible effect on the performance of the data acquisition system. These results are not depicted for brevity. A full torso scan is performed in less than thirty seconds. To obtain a threshold for detection purposes, the phantom with no fluid inside was firstly scanned and the maximum value of the scattered field was defined as the threshold. The obtained images for the upper and lower regions of the healthy phantom are depicted in Fig. 5(a). It should be noted that defining a threshold for healthy cases, specifically for applications targeting clinical tests is by far the most feasible and practical method that has been also successfully utilized for categorizing stroke types^19^. As can be realized, there are high levels of similarity between scanning profiles of lower and higher regions of the torso. To analyze the performance of the system, different amounts of accumulated fluid in different positions were investigated. The phantom was located inside the cavity of the scanner and the water was inserted into three different positions. To that end, 10 mL of water was inserted into a thin and small plastic bag and is positioned in the right side of the phantom behind thorax ribs, in front of the phantom near the skin (shallow target) and towards center of the phantom (deep target). The plastic bag was fixed in the desired positions using thin adhesive tapes. It is noted that the selected positions for water are the ones that could not be detected using the linear scanner^15^. Also, it is worth mentioning that 10 mL is the lowest amount of fluid that a CT-scanner can detect after positioning a patient in a prone position for about half an hour^20^. To magnify the image changes due to presence of fluid (Fig. 5(b)), the scattering profiles of upper and lower regions were subtracted from each other and the resultant images are depicted in Fig. 5(c). The exact location of the fluid is defined by a red circle in all images. As seen, the system is capable of detecting the presence and location of the inserted fluid with a high precision. This is an important capability that provides valuable information regarding the exact position of the accumulation to doctors in cases where invasive sampling is required to define the source of fluid leakage by examining the substances present in the fluid.

In order to define the limitations of the system, the injected fluid amount inside the lungs was reduced to extremely low amount of 3 mL, and was inserted at the previously explained positions. The subtracted images are depicted in Fig. 5(d). Analyzing the images reveals the fact that both detection and precision are dramatically deteriorated by reducing the amount of fluid. In the first case where water is located near the skin, the detected position has a shift of around 50% compared to the original position of the inserted fluid, and in the second situation, where fluid is located behind ribs, a relatively strong scatterer is also detected in addition to the identified fluid. Moreover, the system is not able to detect fluid that is located deep inside the thorax. The main reason behind this phenomenon is the strong reflections from the skin that mask the weak reflections from the small amount of fluid inside the torso. To better understand the aforementioned analysis, the scattering field intensity for 10 mL and 3 mL are plotted in Fig. 5(e). As realized, in the case of 10 mL, the obtained scattering field intensity is well above the maximum scattering intensity obtained from the subtraction of the upper and lower regions in the healthy case, and hence all cases could be precisely detected. However, in the 3 mL case, some regions have a field intensity that falls below the healthy threshold, thus, the level of the received scattered signal is equal or lower than that of a healthy case, and hence no accurate detection can be realized. Therefore, the system with mono-static configuration is limited in terms of detecting low amounts of fluid in early thoracic fluid accumulation.

### Multi-Static Measurements

**Figure 6:**
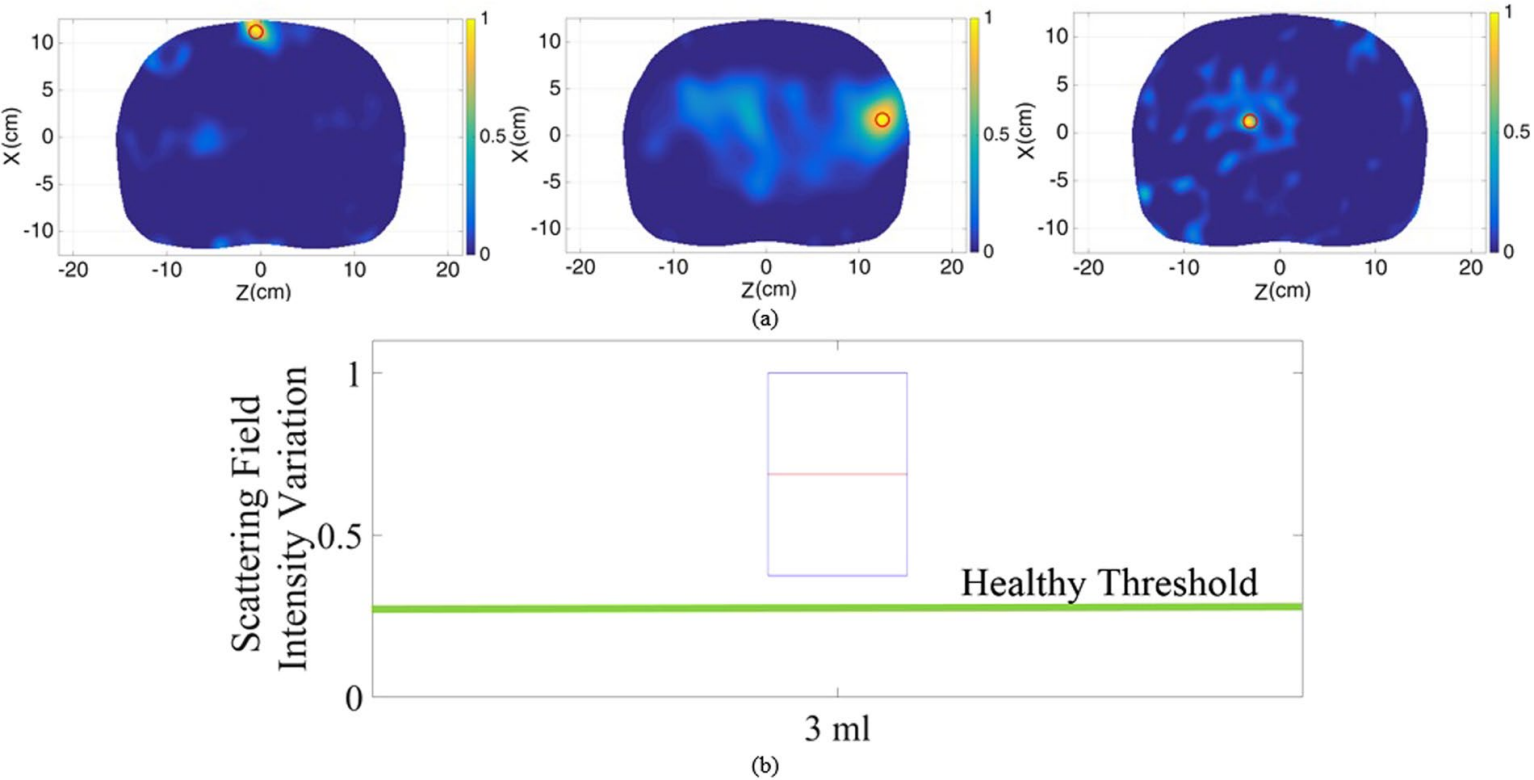
(**a**) Subtracted images of the upper and lower regions at three different positions; front side of the torso left), behind ribs middle) and deep inside thorax right) with 3 mL injected water. (**b**) Scattering field intensity variation diagram in case of 3 mL inserted water compared to the healthy threshold.

To cover the limitations of the mono-static system multi-static scanning approach is devised. In the multi-static system, each antenna array is connected to the switching platform which can sequentially select any antenna to be the transmitter while all the other antennas form the multistatic receiver. To compare the obtained results with those of the monostatic configuration, 3 mL of water was inserted at the same previous positions and measurements were conducted. With the multistatic switching system, it takes less than six minutes, which is obviously longer than the time needed in monostatic scanning, to complete the scanning process. The obtained data is then processed using the aforementioned algorithm to generate the differential images depicted in Fig. 6(a). As can be realized, increasing the amount of the received data from different paths inside the thorax significantly improves the quality of images in addition to the accuracy of diagnosis. It is evident that with the multi-static configuration, the system is capable of the accurate detection and localization of the fluid even when it is small and located deep inside the torso. This is also evident form Fig. 6(b), where the intensity of the back scattered signals are compared to that of healthy one. In contrast to the case with mono-static approach, the intensity of the signals is well above the healthy threshold.

### Human Test Results

**Figure 7:**
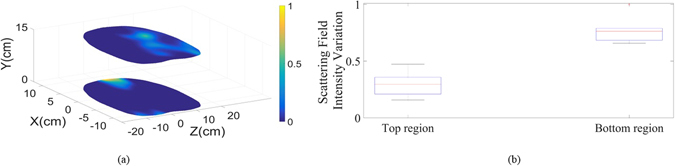
(**a**) Reconstructed images for top and bottom regions of the torso of one of the tested healthy subjects. (**b**) Scattering field intensity variation range for top and bottom regions for all of the six cases.

After confirming capability of the system in detecting small amounts of fluid inside the thorax using phantom, it was tested on healthy subjects as an important step in defining the threshold of image intensity for healthy cases. Defining a range for healthy cases in order to establish a reliable threshold is a well-established technique in clinical applications that has been used for analyzing mammogram^21^ and MRI images^22^. However, it has never been used for torso imaging application. To that end, an informed consent was obtained from all the subjects involved in the study. All of the tests were conducted in accordance with the guidelines and protocols approved by the Ethics Committee of The University of Queensland (Australia). It was assured that the subjects under study do not have any known cardio vascular or related disease. Six subjects with different body sizes and genders were tested. To mimic the realistic situation, the tests were conducted without controlling the body movements, chest movements or breathing cycle control. Moreover, to examine the calibration capability of the system, the tests were conducted during different days and while the system was turned on and off randomly for different cases. These tests were mainly conducted to verify the notion that the upper and lower portions of torso possess fairly similar scattering profiles in addition to investigate the variation range in the image intensity of these two regions with different subjects. Considering that the presence of fluid significantly changes the similarity in the intensity of the scattered field, unhealthy subjects can be identified if their scattering profile is well above the defined range. To de-identify the studied cases, the obtained results from a random human subject is depicted in Fig. 7(a). As can be realized, there is a meaningful similarity between upper and lower regions with no strong scatterers inside the torso. An analysis on the variations between levels of the signals at upper and lower slices of all the cases is depicted in Fig. 7(b). It is realized that the signal levels received from the upper region of the torso are slightly stronger than those from the lower region due to the slightly shorter upper antenna array-body distance than the distance between the lower antenna array. However, the variation between the upper and lower regions of six different cases with different genders and body sizes are in a very confined range. Therefore, this system shows the potential for preclinical tests on a wider range of people and at final stages on subjects with accumulated thoracic fluid.

## Methods

In this section the utilized imaging algorithm is explained. It is noted that the main focus of this paper is the design of the torso scanner system, and data acquisition setup. Thus an existing imaging algorithm is modified and applied to merely verify the imaging capabilities of the proposed platform.

### Imaging Algorithm

To map the recorded backscattered signals to images, a fast frequency-based microwave multistatic imaging algorithm^23^ is utilized. This algorithm uses a two-step beamforming technique to firstly remove the effect of noise and background reflections from the recorded raw data in the pre-processing step and then generating two-dimensional images in the image construction step. Due to the differences between the dielectric properties of the skin and the air in the gap between the antenna and the skin, a portion of the transmitted electromagnetic wave is reflected back to the antenna. This reflection can mask the weak target reflections and hence causes false detections in the imaging process. To address this problem, the average subtraction method in the frequency-domain^23, 24^ is used. In this method, the average value of the collected data (*S*_*avg*_) in an antenna array is subtracted from the value of each antenna (*S* ^*meas*^) at the array at each frequency sample of *f*_*j*_:

1 $${S}_{nm}({f}_{j})={S}_{nm}^{meas}({f}_{j})-{S}_{avg}({f}_{j}),\quad \quad j=1\,{\rm{to}}\,{N}_{f}.$$

where,

2 $${S}_{avg}({f}_{j})=\frac{1}{{N}_{a}^{2}}\sum _{i=1}^{{N}_{a}}\sum _{j=1}^{{N}_{a}}{S}_{ij}^{meas}({f}_{j}),\quad \quad j=1\,{\rm{to}}\,{N}_{f}.$$

In these equations, *N*_*a*_ is the number of antennas and *N*_*f*_ is the number of frequency samples. After eliminating the skin reflections, the image construction algorithm is applied. In the image construction step, the scattering strength of a point scatterer *p* on the *x*-*z* plane of the antenna is estimated by virtually back-propagating the received signals *S*_*nm*_ to the imaging region (see Fig. 4):

3 $${I}_{p}=|\sum _{j=1\,}^{{N}_{f}}\sum _{m=1\,}^{{N}_{a}}\sum _{n=1}^{{N}_{a}}{S}_{nm}({f}_{j}){G}^{\ast }({k}_{j}{r}_{np})|$$

where, *r*_*np*_ is the distance between the *n*th receiver and the point scatterer *p*. In this equation, *G* is the two-dimensional Green’s function of the point scatterer and * denotes the conjugate operator which is equivalent to time-reversal of the signal in frequency-domain. The green’s function *G* at each frequency sample *f*_*j*_ is specified by^23^:

4 $$G({k}_{j}{r}_{np})={J}_{1}^{2}({k}_{j}{r}_{np}){e}^{i2{k}_{j}{r}_{np}}$$

where, *J*_1_(.) is the first kind of Bessel function.

The scattering strength of the point scatterer *I*_*p*_ delivers high values in the location of significant scatterers/target tissues, due to their contrast in dielectric properties with other tissues. To remove the effect of other strong tissues, the values of the point scatterer *I*_*p*_ inside the torso region is calculated for the upper and lower arrays and then subtracted from each other:

5 $${I}_{sub}=|{I}_{low}-{I}_{up}|$$

Due to the similarity of the upper and lower parts of the torso, the subtraction process highlights any changes that may have occurred due to the presence of a target (thoracic fluid).

The imaging algorithm for the monostatic configuration (only one antenna transmits and receives at any time) is extracted from the above explained fast frequency-based microwave multistatic imaging algorithm. In that regard, the average subtraction technique is applied to the monostatic data to remove the strong reflections from the raw data *S*_*n*_^*meas*^:

6 $${S}_{n}({f}_{j})={S}_{n}^{meas}({f}_{j})-{S}_{avg}({f}_{j}),\quad \quad j=1\,{\rm{to}}\,{N}_{f}$$

where,

7 $${S}_{avg}({f}_{j})=\frac{1}{{N}_{a}}\sum _{i=1}^{{N}_{a}}{S}_{i}^{meas}({f}_{j}),\quad \quad j=1\,{\rm{to}}\,{N}_{f}$$

The image construction process for monostatic data is also similar to that of multistatic one, with a difference in the summation process:

8 $${I}_{p}=|\sum _{j=1\,}^{{N}_{f}}\sum _{n=1}^{{N}_{a}}{S}_{n}({f}_{j}){G}^{\ast }({k}_{j}{r}_{np})|,$$

and the final image is calculated by equation (5). The success of the algorithm for mono-static approach is formerly proven for lung cancer detection^24^.

## Conclusion

A microwave torso scanner for the detection, localization and monitoring of thoracic fluid accumulation has been presented. The system is designed in a semi-doughnut chamber shape and is able to accommodate two arrays of twelve antennas to enclose and thus scan the upper and lower regions of the torso. To accommodate two arrays of antennas in the confined space of the torso area, metamaterial theory is applied to the structure of a conventional Yagi antenna to reduce its size by more than 100%. The system is designed and operated based on the notion that the upper and lower regions of the torso, where heart is not present, possess similar scattering profiles. Considering the fact that fluid accumulates at the lower region of the torso due to gravity, it causes dissimilarity for unhealthy subjects and hence can be detected using appropriate microwave imaging techniques. A threshold based on the aforementioned similarity is defined and several sets of tests using monostatic and multistatic data acquisition configurations were conducted both on an artificial phantom and healthy human subjects. It was shown that the system is able to detect and localize fluid volumes as low as 3 mL which indicates early stages of thoracic fluid accumulation. Moreover, the human tests revealed that the intensity of the scattered field from two different regions of the torso are in a confined range for different body sizes and genders, and hence can be utilized as a threshold to define unhealthy cases which are expected to have values well beyond this threshold.

## Electronic supplementary material

Supplementary information
