# Supplementary material for: Novel Microwave Torso Scanner for Thoracic Fluid Accumulation Diagnosis and Monitoring
Source: Sci Rep. 2017 Mar 22;7:304. doi: 10.1038/s41598-017-00436-w (PMC5428468; doi:10.1038/s41598-017-00436-w)
Supplement: Supplementary file 1 — Supplementary information [file 41598_2017_436_MOESM1_ESM.pdf]

Novel Microwave Torso Scanner for Thoracic Fluid Accumulation Diagnosis and Monitoring

S. Ahdi Rezaeieh*, A. Zamani, K.S. Bialkowski, A.M. Abbosh

School of ITEE, The University of Queensland, St Lucia, 4072, Brisbane, Australia

Email: s.ahdirezaeieh@uq.edu.au

Epsilon Negative (ENG)-Loaded Yagi-Antenna

Introduction: To obtain a clear and high quality image using microwave imaging techniques, the targeted imaging domain, torso in this case, should be enclosed by proper antennas to obtain data from all directions around the domain. Moreover, to acquire information at various frequencies and reduce the effect of the surrounding environment, the utilized antennas should be wideband and unidirectional, respectively. While these specifications are easily achievable in applications that operate at high microwave frequencies, it is a challenging task at the lower end of the microwave band at around 1 GHz, which is defined as the optimum operating band for fluid accumulation detection purposes [1]. The size of the antenna at these low frequencies is large, and thus there is a constraint in the number of deployable antennas in the system. If a small number of antennas are used, the quality of the image and its resolution will significantly deteriorate. There are several approaches to address the large size of conventional antennas including the use of three dimensional structure (3-D) and meandering techniques [2-3]. However, these antennas are either limited by high mutual coupling levels if placed close to each other [2] or narrow operating bandwidths [3]. Both of those effects cause a reduction in the image quality and detection accuracy.

The main limitation regarding the sizes of the antennas is the inverse relation between size of an antenna and its operating frequency. To address this limitation, metamaterial structures were proposed in recent years [4-5]. Metamaterial theory is based on the fact that by loading a conventional transmission line (TL), e.g. an antenna, with shunt inductance and series capacitance, the resonance of the antenna can be lowered without increasing the size of the antenna, and hence miniaturized structures can be achieved [5]. This happens due to the excitation of negative and zero wave mode numbers that are independent from the length of the antenna and are not existent in conventional antennas. However, these structures are usually narrow band and omni-directional and thus not suitable for the proposed wideband system. To address these limitations, a Yagi-antenna loaded with epsilon-negative (ENG) metamaterial unit-cell and a folded reflector is proposed in this paper.

Epsilon Negative Loaded Transmission Line Theory: To better explain the operation of the antenna and analyze its resonance mechanism, firstly a brief theory of epsilon-negative metamaterial structures is presented. ENG structures are a subcategory of the negative refractive index (NRI) metamaterials, where the host transmission line is only loaded with a shunt inductance [6] . These structures were proposed to reduce the complexity of loading both series capacitance and shunt inductance at the same time to form a NRI-TL. The equivalent circuit of an ENG metamaterial unit-cell is depicted in Fig. 1 (a). The dispersion relation of this unit-cell is given by [7]:

$$\beta d = \cos^{-1}(1 - \frac{1}{2}\left(\omega^2 LC^2 - \frac{L}{L_0}\right)), \; if \;\; \theta \ll 1 \; and \; \beta d \ll 1 \qquad (1)$$

, where L and C, are per-unit-length inductance and capacitance of the unit-cell with length of d, θ is the electrical length of the unit-cell and L_0 is the loaded shunt inductance.

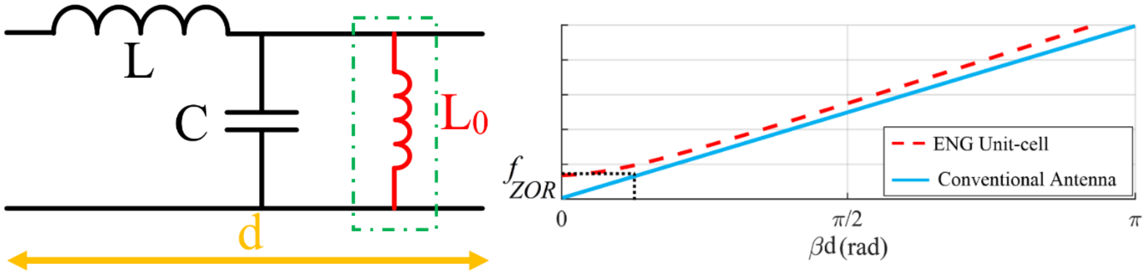

(a) (b)

Fig. 1. (a) Lumped element representation of the equivalent circuit of an epsilon-negative metamaterial unit-cell loaded with shunt inductance (L_0) and its (b) dispersion diagram that shows the zeroth order resonance that is not achievable in conventional configurations.

By utilizing the fact that $\theta = \omega\sqrt{LC}$, the dispersion relation of an ENG-loaded line can be written as [7]:

$$\beta_{ENG} = \sqrt{\omega^2 LC - \frac{L}{L_0}} \quad (2)$$

It can be realized that by simply defining a proper value for the loaded inductance, L_0, zero propagation constant at a desired resonance frequency is achievable ($\omega = 2\pi f$). To verify this process, the dispersion diagram for a unit-cell is depicted in Fig. 1(b). As can be seen, the antenna achieves zero mode number in addition to positive ones and resonance modes can be written as [7]:

$$\beta_{ENG}\, d = \frac{n\pi}{N} \qquad ; n = 0, 1, 2\ldots (N-1) \quad (3)$$

, where N is the number of unit-cells. At zeroth order resonance (ZOR), $n = 0$, the resonance is independent of the size of the unit-cell. This can be clearly seen from Fig. 1 (b), where the dispersion diagram of ENG-loaded unit-cell is compared to that of a conventional antenna. To achieve similar resonance with that of ZOR, a conventional antenna needs to achieve a certain value for β which means the transmission line should have a certain length, while ZOR is independent of the length and resonates when $\beta = 0$. To define a formula for ZOR, the open and short boundary conditions of a conventional transmission line that are defined as [8-9]:

$$Z_{in}^{open} = -jZ_0\, cot(\beta L) \approx -jZ_0 \frac{1}{\beta L} = \frac{1}{NY} \quad (\beta \to 0) \quad (4)$$

$$Z_{in}^{short} = jZ_0\, tan(\beta L) \approx jZ_0 \beta L = NZ \quad (\beta \to 0) \quad (5)$$

, are applied to the unit-cell proposed in Fig. 1(a), and the resultant open boundary conditions are selected. Consequently, ZOR resonance is calculated as [7, 9]:

$$f_{ZOR} = \frac{1}{2\pi\sqrt{L_0 C}} \quad (6)$$

This resonance is independent of the size of the antenna, $l = Nd$, and can be easily lowered by only increasing the value of L_0.

Design Procedure: By utilizing the abovementioned theory, an epsilon-negative loaded Yagi antenna is designed and presented in Fig. 2(a). The antenna is designed on an epoxy FR4 substrate with dielectric constant of 4.4, loss tangent of 0.02 and thickness of 0.8 mm. It is comprised of a folded half-wave dipole as driven element, a folded reflector and a director. It is

fed using a coplanar waveguide (CPW) structure from the center of the folded dipole. The geometrical details of the antenna are presented in Table I. Considering the confined length of a torso of an average human being and the requirement of the system to use two sets of antenna arrays in such a confined space, the side length of the antenna is selected as 120 mm, that is around half of the size required for a conventional Yagi-antenna[10] operating at 0.7 GHz. To excite the size-independent ZOR resonance, the antenna is loaded with ENG metamaterial unit-cells. To realize the loaded inductance, several approaches including the use of chip inductors and via-hole connections can be followed. Yet, these techniques pose manufacturing difficulties or have low inductance values, respectively. To that end, planar microstrip inductors that are connected to virtual grounds were proposed recently [11]. The amount of loaded inductance is defined by the length of the strip and larger values can be obtained using longer strips. Therefore, to achieve lower resonance while keeping the size of the antenna compact, meandered strip inductor is utilized in this work. As mentioned before, the main disadvantage of metamaterial loading is the resultant narrow band. To address this issue, the reflector of the antenna is modified from its conventional setup, which generally is a straight strip with fixed width, to a folded reflector with stepped width. By applying this modification, two main goals are achieved; firstly, the side length of the antenna is kept small by folding operation and secondly the input impedance of the antenna is tuned both by the capacitive coupling created between dipole and the reflector and the stepped width created on the dipole. The director is located at a close vicinity of the dipole to both increase the directivity of the design and secondly enhance the operating bandwidth of the antenna at higher frequencies. As shown in Fig. 2(b), the antenna achieves a wide measured fractional bandwidth of 86% at 0.64-1.6 GHz, which is in a good agreement with simulated results from Ansys Electronic Desktop (AEDT) simulator. The proposed structure is more than 100% smaller, in area size, compared with the reported wideband Yagi-antenna designs [12-14].

The other requirement of the system is the unidirectional radiation from the antennas towards the torso with negligible radiation to other undesired directions, such as backward radiation. This is specifically important due to two main reasons; firstly, the utilized microwave power at medical applications is kept minimal for safety considerations, and thus it is extremely beneficial to focus the radiated power at the desired direction. Secondly, as the proposed portable medical device will not be used in a shielded environment, unidirectional radiation prevents the system to be affected by the surrounding setting such as reflections from walls, and nearby wireless systems. Considering the fact that the human torso is wider at the upper compared to the lower part, side antennas experience larger distances to the body compared to the back and front ones, and thus, both of the near- and far-field patterns should be investigated. As can be seen from the measured results at the sample frequencies of 0.68 GHz, 0.9 GHz and 1.3 GHz (See Fig. 2(c)-(f)), the antenna has a unidirectional radiation at the near- and far-field regions. It is noted that for near field measurements a probe from Aaronia AG©[15] is located at a 1 cm distance from the center of the antenna. To verify the penetration capabilities of the antenna, the whole system is simulated and the resultant electric field inside cross section of torso is plotted in Fig. 3. It is evident that because most of the accepted power is radiated towards the torso area, the front and side antennas achieve high penetration levels, deep inside the torso.

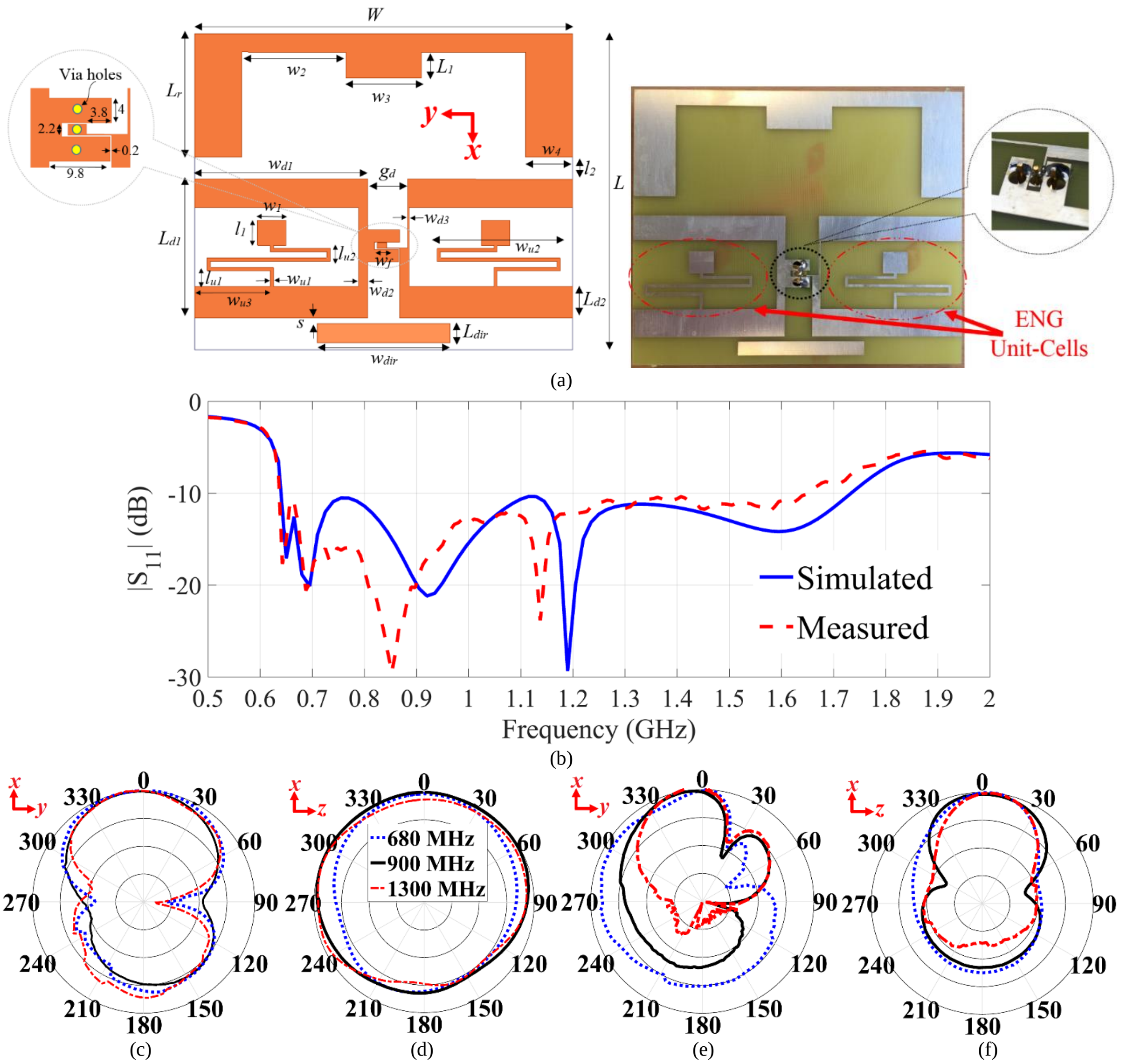

Fig. 2. (a) Geometrical details (left) and prototype of the proposed antenna. The antenna is fed using via holes created on the CPW feeding line by pushing SMA connector through these holes and soldering them to their associated arms. The lower pins of the SMA are removed to enable its access through holes. (b) Simulated vs. measured reflection coefficient of the antenna. (c)-(d) Far-field and (e)-(f) near field radiation patterns of the antenna at sample frequencies of 680 MHz, 900 MHz and 1300 MHz at x-y plane and x-z planes (Identical legends, scale: 40 dB).

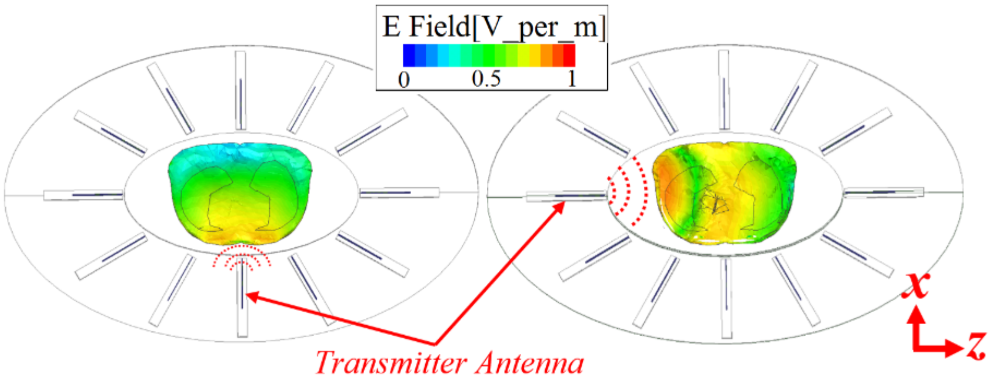

Fig. 3. Cross-sectional electric field distribution inside a torso model due to transmitted signals from the antenna in two different cases where front and side antennas radiate.

1. Rezaeieh, S.A., Abbosh, A., & Wang, Y. Wideband unidirectional antenna of folded structure in microwave system for early detection of congestive heart failure. *IEEE Trans. Antennas Propag.* **62**, 5375-5381 (2014).
2. Mobashsher, A.T., Mahmood, A. & Abbosh, A.M. Portable Wideband microwave imaging system for intracranial hemorrhage detection using improved back-projection algorithm with model of effective head permittivity. *Sci. Rep*. **6**, 20459 (2016).
3. Sabban, A. New wideband printed antennas for medical applications. *IEEE Trans. Antennas Propag.* **61**, 84-91 (2013).
4. Cheng-Jung, L., Leong, K. M. K. H. & Itoh, T. Composite right/left-handed transmission line based compact resonant antennas for RF module integration. *IEEE Trans. Antennas Propag.* **54**, 2283-2291 (2006).
5. Antoniades, M. & Eleftheriades, G. Multiband compact printed dipole antennas using NRI-TL metamaterial loading. *IEEE Trans. Antennas Propag.* **60**, 5613-5626 (2012).
6. Forati, E., Hanson, G. W. & Sievenpiper, D. F. An epsilon-near-zero total-internal-reflection metamaterial antenna. *IEEE Trans. Antennas Propag.* **63**, 1909-1916 (2015).
7. Antoniades, M.A., Mirzaei, H. & Eleftheriades, G.V. *Transmission-line based metamaterials in antenna engineering* (Springer, 2015).
8. Wei, K., Zhang, Z., Feng, Z. & Iskander, M. F. A MNG-TL loop antenna array with horizontally polarized omnidirectional patterns. *IEEE Trans. Antennas Propag.* **60**, 2702-2710 (2012).
9. Caloz, C. & Ito, T. *Electromagnetic metamaterials: Transmission line theory and microwave applications* (John Wiley & Sons, 2005).
10. Dregely, D. *et al.* 3D optical Yagi-Uda nanoantenna array. *Nat. Commun* **2**, 267 (2011).
11. Sharma, S. K., Gupta, A. & Chaudhary, R. K. Epsilon negative CPW-Fed zeroth-order resonating antenna with backed ground plane for extended bandwidth and miniaturization. *IEEE Trans. Antennas Propag.* **63**, 5197-5203 (2015).
12. Bhattacharya, R., Garg, R. & Bhattacharyya, T. K. A compact Yagi-Uda type pattern diversity antenna driven by CPW-Fed Pseudomonopole. *IEEE Trans. Antennas Propag.* **64**, 25-32 (2016).
13. Jiangniu, W., Zhiqin, Z., Zaiping, N. & Qing-Huo, L. Bandwidth enhancement of a planar printed quasi-Yagi antenna with size reduction. *IEEE Trans. Antennas Propag.* **62**, 463-467 (2014).
14. Luo, Y. & Chu, Q. X. A Yagi-Uda antenna with a stepped-width reflector shorter than the driven element. *IEEE Antennas Wireless Propag. Lett.* **15**, 564-567 (2016).
15. *Aaronia AG Product catalogue.* (2014)
    Available: http://www.aaronia.com/Datasheets/Antennas/RF-Near-Field-Probe-Set.pdf. (Accessed: 15th June 2016).

Table I: Geometrical details of the proposed design in (mm).

W	w_1	w_2	w_3	w_4	w_{d1}	w_{d2}	w_{d3}	w_{u1}	w_{u2}	w_{u3}	w_{dir}
120	9	33	24	15	15	3	0e.4	1	39	24	42
L	L_r	L_{d1}	L_{d2}	l_1	l_2	l_{u1}	l_{u2}	L_{dir}	s	g_d	w_f
100	39	44	10	8	7	5	4	6	1.8	12.6	3
